# Flocculation Mechanism and Microscopic Statics Analysis of Polyacrylamide Gel in Underwater Cement Slurry

**DOI:** 10.3390/gels11020099

**Published:** 2025-02-01

**Authors:** Hao Lu, Bo Dai, Chunhe Li, Hua Wei, Jinhui Wang

**Affiliations:** 1 Nanjing Hydraulic Research Institutes, Materials & Structural Engineering Department, Nanjing 210029, China; luhaoxiaobai@163.com; 2 College of Water Conservancy & Hydraulic Engineering, Hohai University, Nanjing 210098, China; 3 Nanjing Hydraulic Research Institutes, Dam Safety Management Department, Nanjing 210029, China; bdai@nhri.cn; 4 Civil and Environmental Engineering, University of Miyazaki, 1-1 Gakuenkibanadainishi, Miyazaki 889-2192, Japan; 5 Jiangxi Academy of Water Science and Engineering, Nanchang 330029, China; wjh_jawse@163.com

**Keywords:** PAM, adsorption, lubrication, entanglement, microscopic statics analysis

## Abstract

Zeta potential testing, Fourier infrared spectroscopy, and total organic carbon analysis were employed in this manuscript to explore the flocculation mechanism of polyacrylamide (PAM) on slurry with a high content of polycarboxylate ether (PCE). Through the combination of assessments of chemical bond shifts, adsorption indicators, and intrinsic viscosity of high-molecular-weight polymer systems, the microscale flocculation mechanisms of different PAM dosages in cement suspensions were elucidated, showcasing stages of “adsorption–lubrication–entanglement”. Initially (PAM < 0.3%), with PAM introduction, the polymer primarily underwent adsorption interactions, including hydrogen bonding between the ester group, amine group, and water molecules; chelation between the ester group and Ca^2+^ and Al^3+^ on the cement surface; and bridging between PAM’s long-chain structure and cement particles. As the PAM content increased, the cement particles’ adsorption capacity saturated (PAM < 0.67%). The entropy loss of polymer conformation could not be offset by adsorption energy, leading to its exclusion from the interface and depletion attractive forces. Slurry movement shifted from inter-particle motion to high-molecular-weight polymer sliding in interstitial fluid, forming a lubrication effect. With further PAM content no less than 0.67%, the polymer solution reached a critical entanglement concentration, and the contact of the rotation radius of the long-chain molecules led to entanglement domination. By introducing bridging adsorption, depletion attraction, and entanglement forces, the cohesion of cement-based polymer suspensions was subsequently determined. The results showed a linear correlation between cohesion and PAM concentration raised to powers of 0.30, 1.0, and 0.75 at different interaction stages, and a multiscale validation from microscopic flocculation mechanisms to macroscopic performance was finally completed through a comparative analysis with macroscopic anti-washout performance.

## 1. Introduction

Polyacrylamide gel is a kind of superabsorbent polymer. Owing to its remarkable properties of viscosity modifying, internal curing [1,2], water solubility [3], and structure tunability [4], PAM gel can be tailored as thickener [5,6], anti-washout admixture [7,8,9], water-retaining agent [3], and shrinkage reducing agent [10,11] in various construction industries, such as ultra-high performance concrete, fiber-reinforced concrete, underwater concrete, 3D printing concrete, etc. In the petroleum industry, Telin [12] found that by adding nano-silicon particles and polyacrylamide to the hydrogel, the elastic modulus was almost doubled, and the ultrasonic shear wave velocity (USS) was almost tripled. Poyraz [13] found that composite hydrogel composed of polyacrylamide (PAAm), starch and gelatin has good swelling properties, attractive optical properties, and a good controlled release mechanism, which provides a feasible way to improve the efficacy of amoxicillin and other drugs. Using low-molecular-weight polyacrylamide as raw material, the crosslinking time-controllable polymer synthesized by Wang [14] can effectively realize the plugging of neural engineering cracks and caves.

Nevertheless, due to the variety of its substructure, molecular weight, and electrical properties, the specific mechanisms of PAM gel in cement-based materials are still not fully understood. Li [15] found that the amide group in the PAM structural unit could easily combine the suspended water molecule, resulting in good water solubility and high chemical activity. In [16], the different charge densities of PAM were studied through adsorption, fluidity, bleeding, and rheology tests. The results demonstrated that anionic and nonionic monomers of the polymer explained the comparable impact on the properties of the fresh cement paste, and the polymer with medium charge density had the strongest effect on the cement paste. After combined solution viscosity, hydrodynamic radii, and adsorption measurement, Bessaies-Bey [1] pointed out that several cement granules could be connected through the absorption of the anionic PAM, resulting in the growth of yield stress values. Hou [17] investigated the reinforcement mechanism of the PAM molecular structure with the hydrogel–cement nanocomposite using molecular dynamics simulation. The results showed that cross-linked structures were formed between the PAM gel and were non-aggregated in the cement nanocomposite with Ca (OH)_2_ nano-spherulites and PAM as the connection nodes.

The role of PAM gel in cement slurry is a complex process involving both physical and chemical interactions. Chemically, PAM gel mainly adsorbs by forming hydrogen bonds between its amino groups and water molecules in the cement slurry. The chain structure of PAM can also chelate with metal cations like Ca^2+^ and Al^3+^ on the surface of cement particles [18]. By Fourier infrared spectroscopy, Simeonov [19] found that there is an interaction between the hydrogel matrix and iron ions through the carboxyl group of polyacrylic acid, and this interaction strongly affects the formation of magnetite particles. Physically, PAM facilitates bridging and entanglement. Bridging occurs as the long-chain structure of PAM allows it to bridge multiple cement particles together [1]. Entanglement refers to the intertwining of PAM molecules within and between each other [12] as well as the entanglement between PAM molecules and cement particles. Long [20] found that the newly synthesized weak gel system had a unique network structure, and with the increase in the concentration of partially hydrolyzed polyacrylamide, the network structure became tighter and exhibited pseudoplastic behavior. Additionally, as the polymer content increased, the influences of adsorption and bridging became more pronounced, leading to a gradual decrease in the fluidity of the slurry. However, contrary to our previous research on anti-washout cement paste, the flowability of the suspension demonstrated a “decrease–increase–decrease” pattern with increasing dosages of the anti-washout admixture, while the corresponding plastic viscosity exhibited an “increase–decrease–increase” pattern [9,21]. The exact effect of PAM gel in anti-washout cementitious environments displays different mechanisms according to the various content.

To address the aforementioned concerns, this study firstly investigated the zeta potential variation mechanism of PAM slurry and integrated previous findings on fluidity and rheology. A main aim was to elucidate the “adsorption–lubrication–entanglement” mechanism of PAM polymer in cement slurry with a high dosage of PCE. By leveraging techniques such as Fourier transform infrared spectroscopy, total organic carbon testing, and theoretical calculations of polymer intrinsic viscosity, the specific actions of the polymer in cement slurry at different dosages were identified. After quantifying the transition point of polymer dosage, a conceptual model of polymer cementitious suspension was constructed. Through employing a modified micro-mechanical theory of fine particle statics, a cross-scale analysis of micro-flocculation mechanisms and macroscopic performance was consequently conducted.

## 2. Results and Discussion

### 2.1. Flocculation Microscopic Mechanism

#### 2.1.1. Zeta Potential Analysis

**Figure 1:**
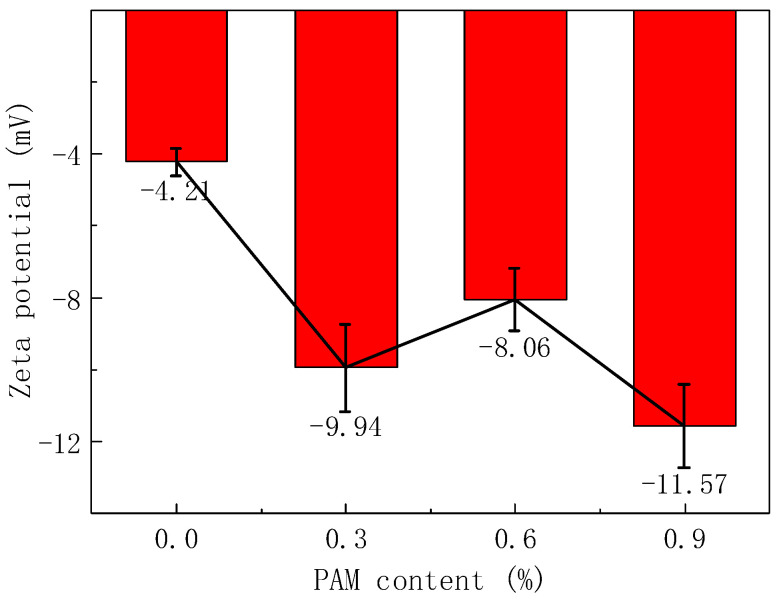
Zeta potential results for pastes with different PAM content.

To investigate the specific behavior of PAM gel in cement slurries, zeta potential tests were conducted on cement pastes with varying PAM contents. As shown in Figure 1, all cement pastes exhibited negative zeta potential values. This is because positively charged ions like Ca^2+^ and Al^3+^ from cement particles dissolve into the aqueous solution, resulting in an abundance of silicate anions on the surface of cement particles and exhibiting negative charge characteristics. When the PAM content was 0.0%, the zeta potential of the cement paste was measured at −4.21 mV, which was lower than that of traditional cement paste [22]. This reduction in zeta potential can be attributed to the electrostatic adsorption of polycarboxylate superplasticizer molecules on the surface of cement particles. These molecules neutralize some of the surface charges, weakening the electrical properties and decreasing the absolute value of the zeta potential. Zeta potential serves as an essential indicator of colloidal stability. Higher absolute values indicate greater stability, while values closer to zero suggest a tendency for particles to aggregate. In this case, the decrease in the slurry’s zeta potential leads to the sedimentation of cement particles, resulting in observable macroscopic phenomena such as bleeding and segregation, which align with practical observations.

With the addition of PAM to the cement paste, its adsorption through chemical bonding and bridging due to its long-chain structure stabilized the spacing between cement particles, reducing the occurrence of particle aggregation and adhesion. This results in improved stability of the cement paste and an increase in the absolute value of the zeta potential. As the PAM content increased, the available adsorption sites on cement particles gradually decreased. Until the PAM content approached 0.3%, both PAM and PCE reached their saturation adsorption points, and the absolute value of the zeta potential peaked at 9.94 mV. Beyond this point, any excess PAM polymer dissolved in the interstitial liquid of the cement paste as a pure solution. Within this range, the behavior of PAM is similar to that of non-adsorbing polymers, leading to a phenomenon known as “depletion attractive forces”, as explained by Bessaies-Bey [23]. These forces cause cement particles to come closer to each other, resulting in a decrease in the zeta potential. The change in zeta potential due to the presence of depletion attractive forces is relatively small. When the PAM content reached around 0.6%, the zeta potential of the PAM paste reached 8.06 mV. Further increasing the PAM content resulted in an additional increase in the zeta potential. This may be attributed to the gradual dominance of the entangling effect of PAM in the suspension. By acting as entangling nodes around cement particles or by wrapping around them, PAM greatly reduces the adhesion caused by pressure differences between cement particles. This displacement of the slip plane towards the particle surface [24] leads to an increase in the internal stability of the cement slurry and consequently an increase in the zeta potential.

The above analysis regarding the specific behavior of polyacrylamide gel in cement paste correlates well with its macroscopic flowability and plastic viscosity [20]. When the PAM content was low, the adsorption and bridging actions contributed to an increase in the absolute value of the zeta potential, resulting in reduced flowability. As the PAM content increased, it exhibited characteristics similar to those of non-adsorbing polymers, leading to a slight improvement in flowability. However, as the PAM content continued to rise, the entanglement between the large polymer molecules became the dominant effect, causing an increase in the absolute value of the zeta potential and a subsequent decrease in flowability. These different PAM contents demonstrate the existence of “adsorption–lubrication–entanglement” mechanisms in cement slurries. To further validate the accuracy of this mechanism, additional tests such as Fourier transform infrared spectroscopy (FTIR), total organic carbon (TOC) analysis, and critical concentration theory calculations were performed.

#### 2.1.2. Fourier Transform Infrared Spectroscopy (FTIR)

**Figure 2:**
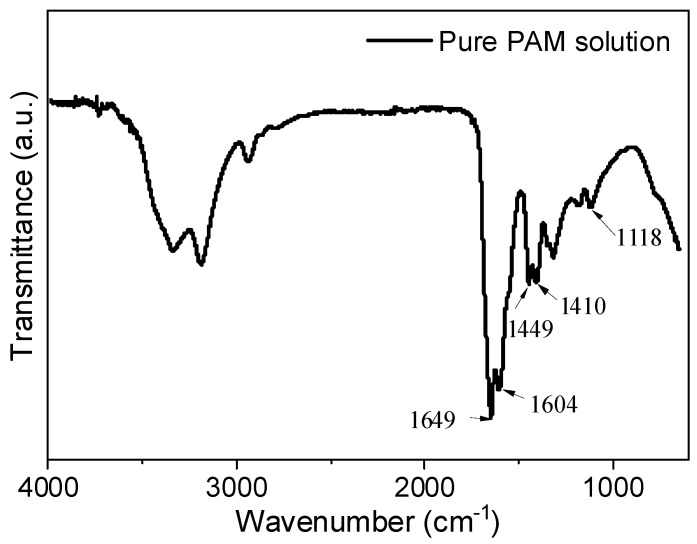
FTIR results of PAM aqueous solution.

**Figure 3:**
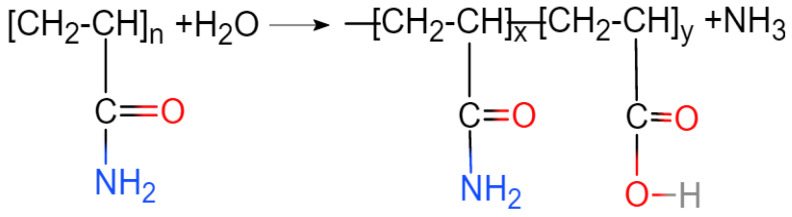
Hydrolysis chemical formula for PAM molecule.

Figure 2 illustrates the FTIR results of a pure PAM solution, where specific peaks are marked at 1649 cm^−1^, 1449 cm^−1^, 1410 cm^−1^, and 1118 cm^−1^. As mentioned in Section 2.1, the primary functional group in PAM molecules is the -CO-NH_2_ group. The stretching vibration of the C=O bond in the -CO-NH_2_ group appears prominently at 1649 cm^−1^ [25,26]. The in-plane oscillation of the amine group (-NH_2_) is observed at 1118 cm^−1^, while the bending vibration of the N-H chemical bond in the amine group is represented by the peak at 1604 cm^−1^ [25]. Furthermore, distinct peaks at 1449 cm^−1^ and 1410 cm^−1^ are observed in the FTIR spectrum. According to research [6], these peaks correspond to the symmetric stretching vibration of the characteristic functional group COO-. This suggests that non-ionic polyacrylamide undergoes hydrolysis in an aqueous solution, resulting in the formation of carboxylic acid functional groups, as indicated in Figure 3.

**Figure 4:**
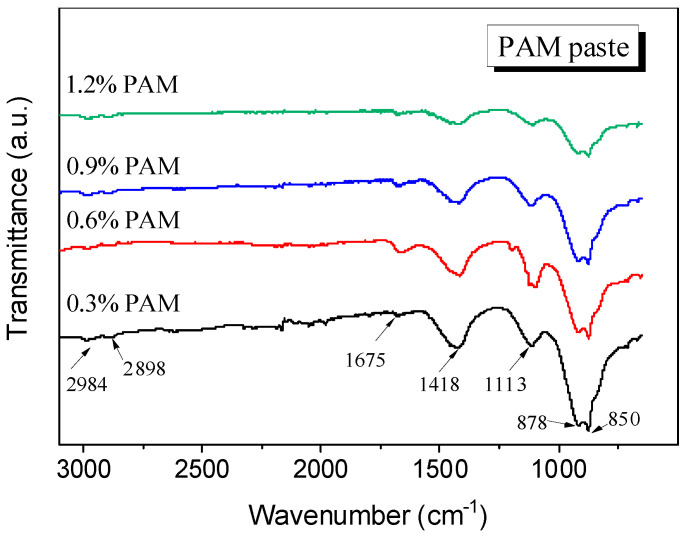
FTIR results of paste with different PAM content.

The FTIR results of the PAM paste are presented in Figure 4, where several characteristic peaks are identified and labelled at 1675 cm^−1^, 1418 cm^−1^, 1113 cm^−1^, 878 cm^−1^, and 850 cm^−1^. The peak at 1418 cm^−1^ corresponds to the symmetric vibrations of the -COO- functional group, while the peak at 1113 cm^−1^ represents the in-plane bending vibrations of the -NH_2_ functional group. The CO_3_^2-^ functional groups of CaCO_3_ present in the cement slurry exhibit out-of-plane and in-plane bending vibrations, resulting in distinct peaks at 878 cm^−1^ and 850 cm^−1^ in the FTIR spectrum, respectively. Furthermore, the absorption peak of hydroxyl groups (-OH) in water molecules can be observed around 1675 cm^−1^. Upon adding PAM gel to the cement slurry, the symmetric stretching vibration peak of -COO- at 1449 cm^−1^ disappears. This disappearance can be attributed to the complexation of -COO- with metal cations on the cement surface, such as Ca^2+^ and Al^3+^, forming chelation interactions [27]. Another characteristic peak of -COO-, originally observed at 1410 cm^−1^ in the pure PAM solution, shifts to 1418 cm^−1^ in the PAM-added paste suspension. Similarly, the -NH_2_ characteristic peak of PAM shifts from 1118 cm^−1^ to 1113 cm^−1^. By considering these observations along with the presence of the -COO- peak at 1675 cm^−1^ in the PAM paste, it can be concluded that there are hydrogen bonding interactions between the -COO- and -NH_2_ groups of PAM and water molecules. This interaction results in a shift of the characteristic peaks, indicating the formation of hydrogen bonds.

**Figure 5:**
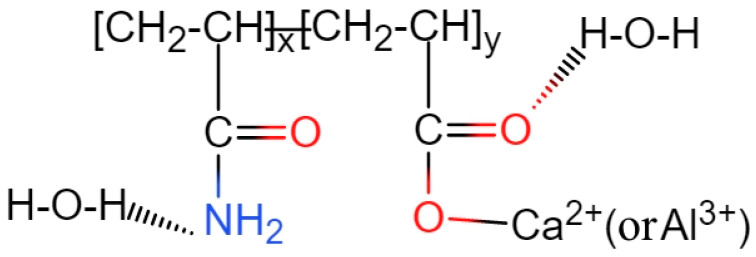
Chemical formulas of PAM added pastes.

Based on the findings, nonionic PAM in the cement slurry exhibits two types of interactions: chelation and hydrogen bonding. The chelation mainly occurs between the -COO- functional groups of PAM and metal cations, such as Ca^2+^ and Al^3+^. This leads to the adsorption of the long-chain structure of PAM onto the surface of cement particles, while hydrogen bonding interactions take place between the -COO-/-NH_2_ and water molecules. These interactions contribute to a reduction in the fluidity of the material, as evidenced by decreased water flow properties. The chemical interactions involving PAM in the cement slurry can be represented by the diagram shown in Figure 5. Combined with the previously established physical bridging interactions between large polymer chains, it forms the primary adsorption form of nonionic PAM in the cement paste suspension.

#### 2.1.3. Total Organic Carbon Test (TOC)

**Figure 6:**
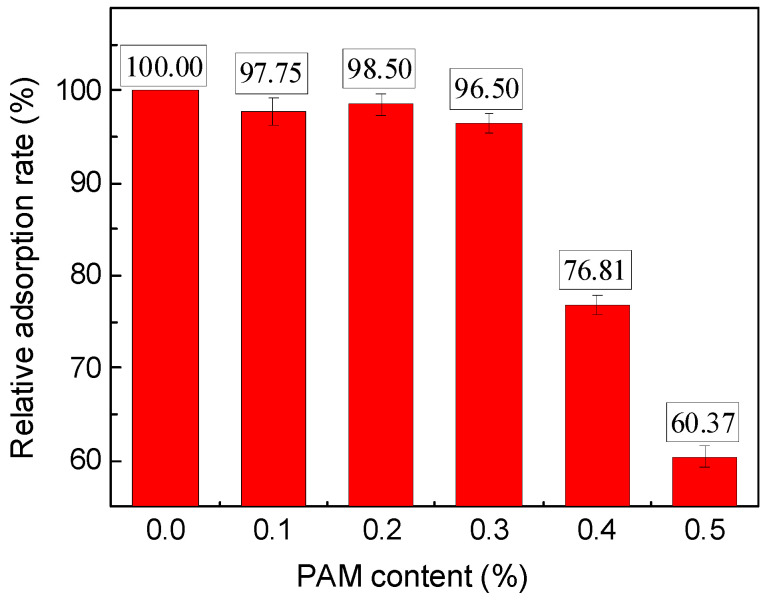
TOC test results of pastes with different PAM contents.

The total organic carbon test results for the anti-washout cement paste at different PAM contents are presented in Figure 6. When the PAM content was 0.0%, the TOC_P+C_ value was found to be zero, indicating a 100.0% solution adsorption rate. As PAM was added to the cement slurry, there was a slight variation in this value, but overall, it remained above 95.00%. This suggests that the cement particles have the ability to adsorb freely available surfaces, allowing for a high relative adsorption rate of PAM’s long-chain structures onto particle surfaces. However, this phenomenon significantly decreased when the PAM content exceeded 0.3%. The relative adsorption rate dropped from 96.50% for a 0.3% PAM content to 60.37% for a 0.5% PAM content. The reason behind this decline can be attributed to the gradual reduction in free surfaces on the cement particles as the PAM content increases. This reduction is not noticeable when the PAM content is low. Once the content reached a saturation point for adsorption, where the polymer completely adheres to the surfaces of cement particles, further increases in PAM content resulted in excess large molecules dissolving in the interstitial liquid. As a result, when the system was centrifuged and separated from the suspension, the TOC_P+C_ value increased, indicating the presence of more dissolved PAM, while the R_a_ value decreased. From these observations, it can be concluded that in a cement slurry with a water-to-cement ratio of 0.5 and a superplasticizer content of 0.2%, a PAM content of 0.3% reaches the saturation point for adsorption on the surfaces of the suspended particles.

#### 2.1.4. Critical Concentration Theory

**Figure 7:**
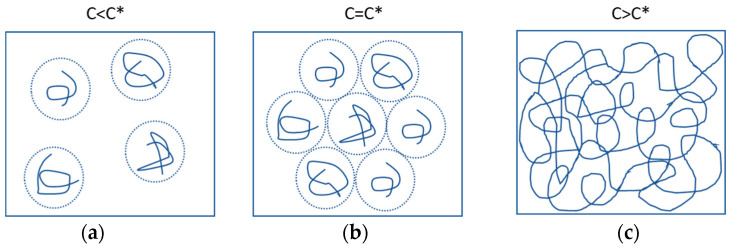
Polymer solution concentrations greater than, equal to, and less than critical concentration: (**a**) c < c*; (**b**) c = c*; (**c**) c > c*.

Polymers in a solution can be categorized into three different concentrations: diluted solution, semi-diluted solution, and concentrated solution. The critical concentration (c*) serves as the threshold between diluted and semi-diluted solutions. In a diluted solution, when the polymer concentration is below the critical concentration, the individual polymer monomers are independent of each other. This means that the polymer molecules behave as separate entities in the solution. As the polymer concentration gradually increases and reaches the critical concentration, the polymer chains start to entangle and interact with each other, forming a semi-diluted solution [28]. In the case of polyacrylamide gels, due to hydrogen bonding, individual polymer molecules often self-entangle, creating specific colloidal structures with hydrodynamic radii. If these colloidal structures are idealized as spherical, the critical concentration corresponds to the point where the hydrodynamic radii of polymer molecules in solution touch but have not begun to interact. Once the polymer concentration exceeds c*, the hydrodynamic radii of colloids start to overlap, and interactions between polymers gradually dominate the system, forming a network of entanglements, as shown in Figure 7.

The specific value of the critical concentration c* in a polymer solution can be estimated approximately using the intrinsic viscosity ([*η*]) of the polymer. According to research [29], the exact relationship between these two parameters can be described by Equation (1):

c* ≅ 2.5/[*η*] (1)

To calculate the critical concentration value for PAM slurry, it is necessary to determine the intrinsic viscosity of the polymer. The intrinsic viscosity can be calculated using the following equation [30]:

(2) $$\eta_{sp}=\left[\eta \right]+k_{1}{\left[\eta \right]}^{2}c+k_{2}{\left[\eta \right]}^{3}c^{2}+\cdots$$

where [*η*] is the intrinsic viscosity of the polymer, *c* is the concentration of the polymer, *k_i_* are constants, and *η_sp_* is the specific viscosity. The specific viscosity is obtained by subtracting 1 from the relative viscosity, as shown in Equation (3):

(3) $$\eta_{sp}=\eta_{rel}-1$$

where *η_rel_* is the relative viscosity, which quantifies the change in liquid viscosity caused by the addition of high-molecular-weight polymers. In polymer blends, the relative viscosity represents the ratio of the viscosity of the dispersed phase to that of the continuous phase, indicating the difference in viscosity between the two phases:

(4) $$\eta_{rel}=\mu_{P}/\mu_{w}$$

where *μ_p_* is the viscosity of the polymer solution, and *μ_w_* is the viscosity of the pure solvent. At room temperature (25 °C), the viscosity of the pure solvent *μ_w_* is taken as 0.8934 × 10^−3^ Pa·s.

By combining Equations (1)–(4), the final formula for calculating the critical concentration can be derived:

(5) $$1.1193\times 10^{3}\mu_{P}=\frac{2.5}{c^{*}}+k_{1}{\left(\frac{2.5}{c^{*}}\right)}^{2}c+k_{2}{\left(\frac{2.5}{c^{*}}\right)}^{3}c^{2}+\cdots$$

Assuming the measured polymer concentration is sufficient, the higher-order terms in Equation (5) can be neglected. This allows for the approximation of a quadratic linear equation to determine the intrinsic viscosity of the non-ionic polymer solution, resulting in the following formula for the critical concentration:

(6) $$2.5k_{1}c=0.448\times 10^{3}{{{(c}^{*})}^{2}\mu }_{P}-c^{*}$$

By substitute the data in [19] into Equation (6), the critical concentration of non-ionic polyacrylamide coagulant is calculated as c* = 0.73 g/dL, which corresponds to 0.67% PAM content in the cement slurry. This implies that the PAM gel indeed undergoes significant entanglement at around 0.6% content, leading to an increase in the absolute value of the zeta potential and a subsequent reduction in the material’s macroscopic fluidity.

From a volumetric dimension perspective, the overlap concentration of a PAM solution is equal to the volume occupied by a PAM coil per unit volume. The critical concentration can also be calculated using the following Equation (7):

(7) $$c^{*}=M_{w}/N{R_{G}}^{3}$$

where *M_w_* is the molar mass of the polymer, *N* is Avogadro’s number, and *R_G_* is the polymer’s radius of gyration. The formula for the radius of gyration is as follows:

(8) $$R_{G}=K_{R_{G}}M_{W}^{\upsilon }$$

where *K_RG_* and *υ* are constants representing the basic properties of the polymer. Based on references [1,9], the values of Avogadro’s constant *N*, *K_RG_*, and *υ* are determined as 6.02 × 10^23^, 0.0074, and 0.6, respectively. Based on this method, the critical viscosity value of PAM with a molecular weight of 10 million is calculated to be reached at a concentration of 0.65%. The value of c* calculated from empirical formulas closely matches the calculation results from Equation (6), with a relative error of only 0.02%. This discrepancy is negligible compared to the 0.1% PAM gradient increment used in this study.

Compared to empirical hydrodynamic radius calculation formulas, the traditional intrinsic viscosity method uses experimentally obtained plastic viscosity values to calculate the critical viscosity value of polymers, making it more accurate and reliable. Therefore, the critical viscosity value of PAM in anti-dispersion slurry was determined to be 0.67%. Once the PAM dosage reaches or exceeds this value, the PAM paste transitions from a dilute solution to a semi-dilute solution, where the entanglements between polymer long chains gradually dominate.

### 2.2. PAM Mechanism Concept Model

#### 2.2.1. Adsorption Mechanism

**Figure 8:**
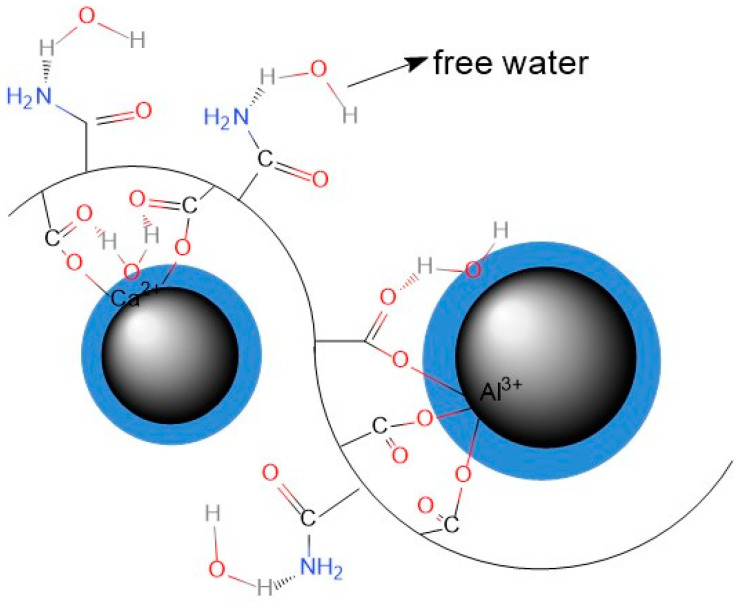
Schematic diagram of PAM adsorption in cement slurry.

When initially added to cement slurry in small amounts, PAM molecules exhibit minimal entanglement due to their low content. Instead, they primarily exist in the suspension system in an adsorbed form. As identified by Fourier transform infrared spectroscopy analysis, this adsorption occurs through two main mechanisms: chelation and hydrogen bonding. The COO- groups in PAM molecules undergo chelation with metal cations (Ca^2+^, Al^3+^), and hydrogen bonding forms between COO-/-NH_2_ groups and water molecules in the PAM solution. The hydrogen bonding is not limited to the thin water film on particle surfaces but also extends to the free water in the interstitial fluid. In addition to these two forms of action, due to the relatively large molecular weight of the polyacrylamide gel used in this study, its extended long-chain structure allows it to simultaneously adsorb onto multiple cement particle surfaces, exhibiting the bridging effect. The concept model for polymer adsorption in cement slurry with low PAM content is depicted in Figure 8.

In this model, the complexation and bridging interactions between PAM and cement particle surfaces directly restrict the movement of the particles. The hydrogen bonding in the cement slurry could decline the lubrication factor needed for particle movement, also leading to the decline of the material’s fluidity. As the PAM content increases within the range below the saturation adsorption capacity, all adsorption interactions within the cement slurry intensify. This leads to a gradual shift of the sliding planes towards the cement particles, resulting in an absolute increase in zeta potential from 4.21 mV to 9.94 mV. This is manifested by an increase in plastic viscosity from 4.26 Ps·s to 9.24 Ps·s and a decrease in fluidity from 22.55 cm to 18.20 cm [19].

#### 2.2.2. Lubrication Mechanism

As the content of PAM increases, the adsorption on particle surfaces increases, resulting in a decrease in the remaining available free surface area for adsorption. The total organic carbon test conducted indicates that when the PAM content reaches 0.3%, its adsorption capacity reaches saturation. At this point, the cement particle surfaces are fully covered by adsorbed PAM molecules, and further addition of the polymer does not result in additional adsorption on the particles. Instead, the excess polymer dissolves in the interstitial fluid of the cement slurry, leading to non-adsorptive polymer traits [23] and causing the depletion attractive forces. The presence of depletion attractive facilitates the approaching of cement particles, resulting in a decrease in the absolute value of the zeta potential from 9.94 mV to 8.06 mV.

**Figure 9:**
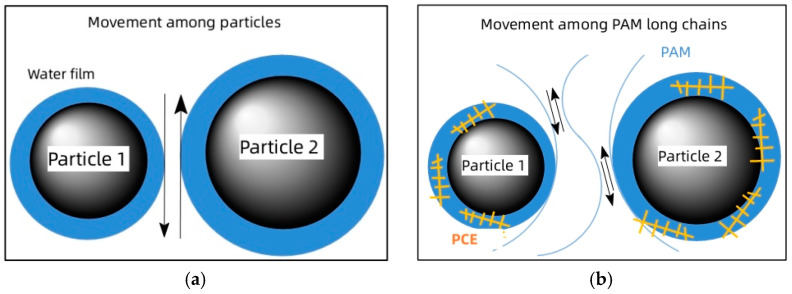
Particle movement mode under pure cement paste (**a**) and PAM lubrication (**b**).

In the absence of PAM, the flow of cement slurry primarily involves the movement of cement particles, with a water film covering the particle surfaces to reduce hindrance and ensure overall fluidity, as depicted in Figure 9a. However, once PAM is introduced, the adsorption process reduces the available free-water volume in the slurry. This limits the water film and also adds constraints to particle movement through bridging effects. Consequently, the interparticle movement decreases significantly. When PAM reaches the saturation adsorption point, due to competitive adsorption between polymer molecules, the cement surface is completely covered by PAM or PCE molecules, along with the adsorbed water molecules. This tremendously restricts interparticle movement; the movement within the cement slurry transforms into the relative motion of high-molecular-weight polymers in the interstitial fluid [31], as depicted in Figure 9b. Within this range, PAM exhibits lubricating properties, and its addition leads to a slight increase in cement slurry fluidity and a corresponding decrease in plastic viscosity.

#### 2.2.3. Entanglement Mechanism

According to the intrinsic viscosity theory, when the PAM content reaches 0.67%, the polymer solution reaches its critical concentration, causing the large long-chain structures of the polymer to interact and entangle with each other. This entanglement structure further wraps around the polymer adsorbed on the cement particle surfaces, resulting in the sliding surfaces in the slurry moving closer to the particle surfaces. This enhances the stability of the polymer system, leading to an increase in the absolute value of the zeta potential of the cement slurry from 8.06 mV to 11.57 mV. As the PAM content continues to increase beyond 0.67%, the flocculation and entanglement within the cement slurry progressively intensify, resulting in a decrease in the flowability of the slurry from 21.05 cm to 18.95 cm [22].

**Figure 10:**
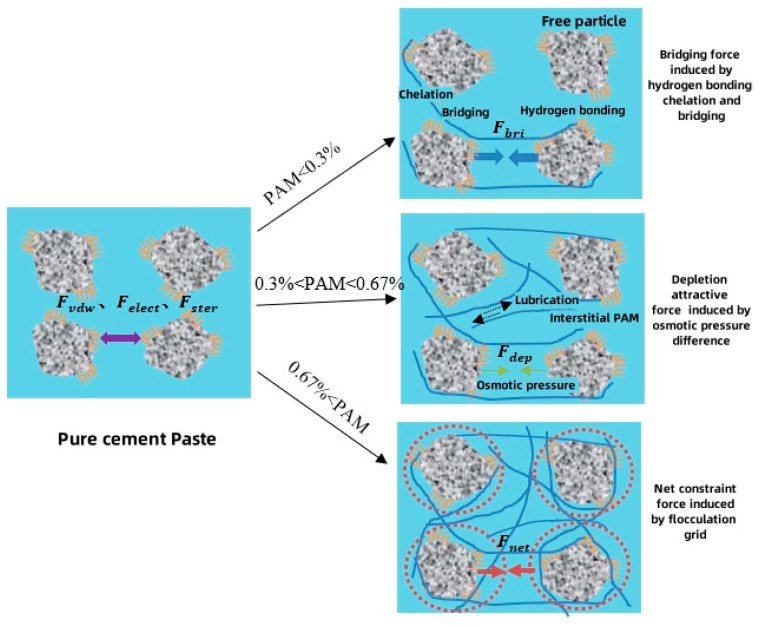
Mechanism model diagram of materials with different PAM content.

To summarize, the determined mechanism model of PAM in cement slurry is depicted in Figure 10. At relatively low PAM content, the adsorption effect of PAM primarily occurs through its long-chain structure in the slurry. However, during this stage, some cement particles and water molecules remain free. Directly performing underwater casting of the PAM slurry in such environment can lead to significant dispersion due to the presence of free cement particles, resulting in poor anti-dispersion performance. As the PAM content increases to its saturation adsorption capacity, a majority of cement particles in the slurry are adequately adsorbed and bridged. Water molecules are also absorbed and encapsulated through hydrogen bonding. Along with the lubricating effect of PAM, the flowability of the slurry gradually improves, and the conditions for underwater casting progressively enhance. When the PAM content reaches the critical concentration of 0.67%, the flowability of the slurry reaches its peak value, and the anti-dispersion performance also achieves a relatively high level. Further increasing the PAM content leads to the formation of a network entanglement structure, resulting in a reduction in flowability.

#### 2.2.4. Model Verification

Due to its characteristics of high early strength and strong bonding strength, CSA cement has significant applications in underwater non-dispersive cementitious materials [32]. To assess the general applicability of determined PAM mechanisms in cementitious materials, the corresponding performance of underwater non-dispersive sulfoaluminate cement was tested in this section.

##### Workability Performance of PAM-CSA Slurry

**Figure 11:**
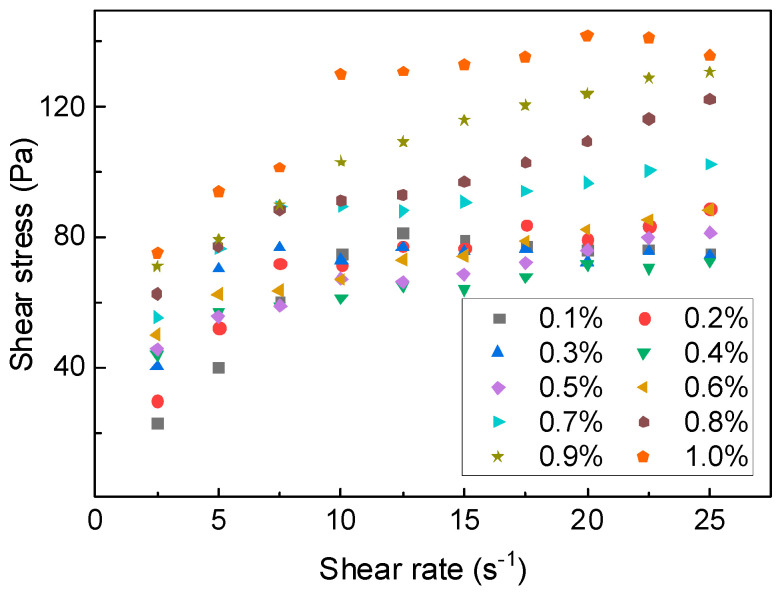
Curve of CSA shear rate and shear stress.

The rheological curve of anti-dispersive CSA cement is presented in Figure 11. It can be observed that the addition of PAM gel induces similar rheological characteristics in sulfate aluminate cement as in ordinary Portland cement. Specifically, the presence of PAM in the slurry causes significant shear-thinning behavior, which gradually weakens with increasing PAM content. Shear thinning occurs as the bonds between material products and the floc-like structure are disrupted during shearing. On the other hand, PAM in the neat slurry can adsorb water molecules and particles or undergo molecular entanglement during shearing. This process results in the continuous destruction and reformation of the internal structure of the suspension. As the PAM content increases, this restorative effect becomes more prominent, leading to an increase in the yield pseudoplastic index.

**Figure 12:**
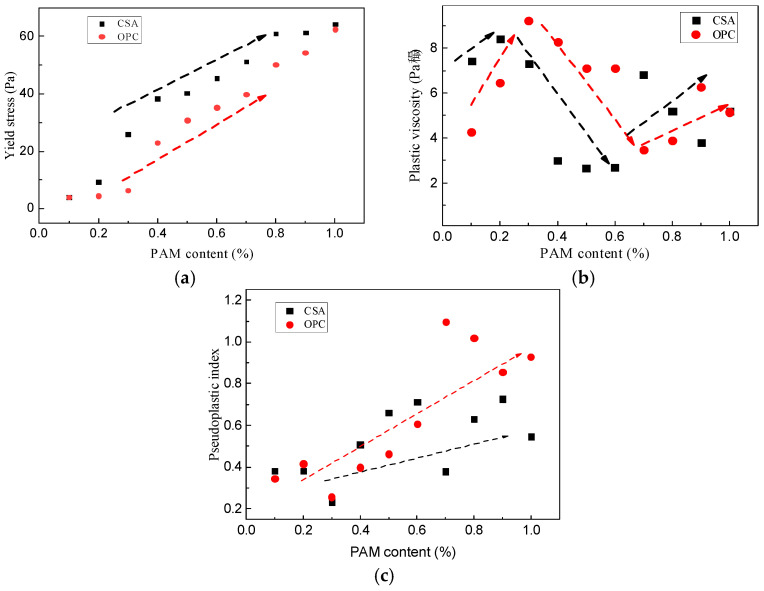
Rheological parameters between CSA and OPC cement under PAM: (**a**) yield stress; (**b**) plastic viscosity; (**c**) pseudoplastic index.

Based on the B-HB model, parameters such as yield stress, plastic viscosity, and apparent yield pseudoplastic index were simulated and analyzed for CSA slurry. The results are presented in Figure 12. For the same PAM content, the yield stress of CSA is higher than that of OPC [19], and the yield stress of CSA increases with higher PAM content. This difference is attributed to the higher content of metal cations in CSA, making it more susceptible to complexation reactions, which enhance its adsorption capacity and yield stress [33]. As shown in Figure 12b, the plastic viscosity of PAM–sulfate aluminate neat slurry also follows an “increase–decrease–increase” trend, similar to the variation pattern of OPC cement. When PAM < 0.2%, the plastic viscosity of CSA increases from 7.431 Pa·s to 8.398 Pa·s; when 0.2% < PAM < 0.6%, the plastic viscosity decreases from 8.398 Pa·s to 2.699 Pa·s; when PAM > 0.6%, the plastic viscosity increases again from 2.699 Pa·s to 5.200 Pa·s. Based on the analysis above, this could relate to the different mechanisms exhibited by PAM at different stages. With the addition of PAM, the CSA material exhibits shear-thinning behavior (i.e., *n* < 1), which is related to the gradual breaking of bonds and the continuous destruction of the floc-like structure during shearing. With increasing PAM content, the re-adsorption and re-entanglement of the long-chain structure in the aqueous environment are enhanced, resulting in an increased restorative ability of the material under shearing conditions and an increase in the apparent yield pseudoplastic index.

**Figure 13:**
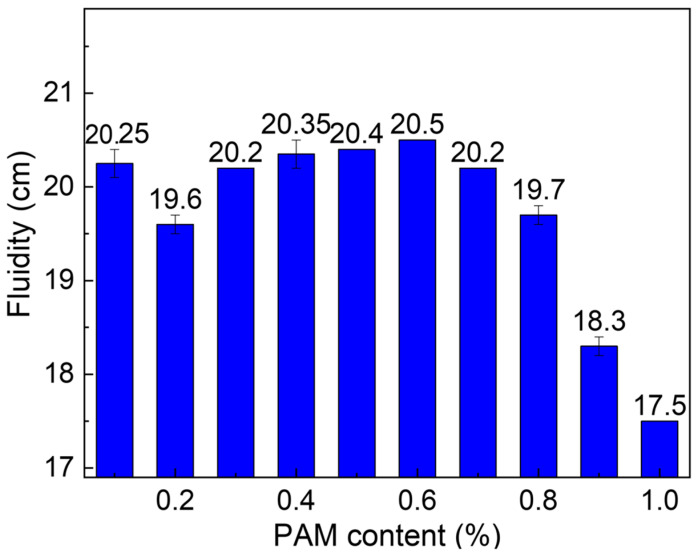
Fluidity results of CSA cement under PAM.

The variation curve of fluidity in sulfate aluminate cement slurry at different PAM contents is depicted in Figure 13. It is evident that the fluidity of the cement slurry follows a pattern of initial decrease, subsequent increase, and then decrease again as the PAM content increases. When the PAM content is less than 0.2%, the fluidity decreases from 20.25 cm to 19.6 cm with increasing PAM content. In the range of 0.2% < PAM < 0.6%, the fluidity of the cement slurry increases from 19.6 cm to 20.50 cm. For PAM content exceeding 0.6%, the fluidity decreases from 20.50 cm to 17.5 cm. The trend of fluidity variation and the critical content points align closely with the values of plastic viscosity.

##### PAM-CSA Slurry Flocculation Mechanism

**Figure 14:**
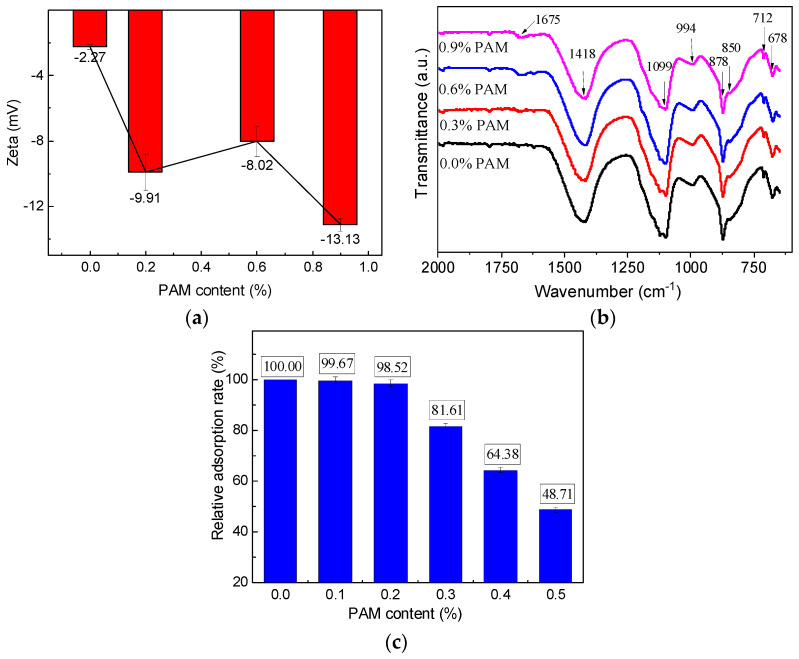
Zeta potential (**a**), FTIR (**b**), and TOC (**c**) results for CSA cement pastes—with 0.0%, 0.3%, 0.6%, and 0.9% PAM.

The FTIR results of sulfate aluminate cement under different PAM dosages are presented in Figure 14b. In the pure PAM solution, the symmetric stretching vibration of -COO- at 1449 cm^−1^ disappears, indicating the complexation of -COO- with metal cations (such as Ca^2+^ and Al^3+^) on the cement surface [27,28]. Another characteristic peak of -COO- shifts from 1410 cm^−1^ in the pure solution to 1418 cm^−1^ in the cement slurry suspension. Similarly, the characteristic peak of -NH_2_ in PAM shifts from 1118 cm^−1^ to 1099 cm^−1^, suggesting the presence of hydrogen bonding interactions between -COO-/-NH_2_ groups and water molecules. This interaction leads to a redshift or blueshift in the peaks corresponding to the functional groups. Since the average particle size of CSA cement is smaller than that of ordinary Portland cement, the bridging effect also occurs in the cement slurry. The combined presence of hydrogen bonding, complexation, and bridging adsorption reduces particle aggregation, enhances the stability of the cement slurry system, and increases the absolute value of the zeta potential from 2.27 mV to 9.91 mV, as shown in Figure 14a.

When the content of PAM is low, there is sufficient residual surface area on the CSA particle surface, and all polymer structures can adsorb onto the cement particles. As the PAM content increases, the amount of free surface available for adsorption on the cement particle surface gradually decreases. According to the total organic carbon test in CSA suspension (Figure 14c), when the PAM content is around 0.2%, the particle surface reaches its saturation loading value. With further increase in PAM content, the polymer will dissolve in the interstitial fluid of the cement slurry as a pure solution. In this case, the cement particles tend to approach and aggregate due to the pressure difference, resulting in a decrease in the absolute value of the zeta potential.

Based on Equation (6), the critical concentration of CSA cement slurry was theoretically calculated from a concentration perspective, resulting in a critical concentration of c* = 0.702 g/dL, equivalent to a PAM content of 0.62% in sulfate aluminate cement. When the PAM content reaches 0.62%, it transitions from a dilute solution to a semi-dilute solution in CSA cement slurry, and the interaction between long-chain polymer structures through mutual entanglement becomes the predominant effect. The slipping of cement particles towards the particle surface leads to an increase in zeta potential from 8.02 mV to 13.13 mV.

Through the aforementioned experimental analysis, it was demonstrated that the role of PAM in sulfate aluminate cement follows the same principles as OPC cement, including the three stages of “adsorption–lubrication–entanglement”. However, due to differences in particle composition and particle size distribution, the transition points of PAM concentration effects may vary. When the PAM content is less than 0.2%, the addition of the polymer primarily causes an adsorption effect, forming hydrogen bonding, chelation, and bridging interactions. This reduces material bleeding, enhances the internal aggregation capacity of the suspension, and increases plastic viscosity while decreasing macroscopic fluidity. In the range of 0.2% < PAM < 0.62%, the addition of the polymer functions primarily as a lubricating agent. It saturates the cement particle surfaces, with PAM existing in the form of a pure solution. This transition leads to a shift in the flow pattern of the suspension from relative movement between cement particles to relative movement between polymers. As a result, plastic viscosity decreases and macroscopic fluidity increases. When the PAM content exceeds 0.62%, it surpasses the critical concentration c*, and the dominant effect becomes the entanglement of high-molecular-weight polymers. This enhances the stability and internal cohesive forces of the suspension system, resulting in increased plastic viscosity and reduced macroscopic fluidity.

### 2.3. PAM Paste Microscopic Statics Analysis

#### 2.3.1. Interparticle Forces

Due to the “adsorption–lubrication–entanglement” effect of PAM gels, the form and magnitude of the interparticle forces between cement particles change. This alteration enhances the overall flocculation and aggregation strength of the suspension, ultimately improving the macroscopic anti-dispersion performance of the material. Therefore, quantitatively analyzing the magnitude of interparticle forces within PAM cement paste is of significant theoretical importance for elucidating its anti-dispersion mechanism.

In this study, all the measurements were conducted directly after mixing the cement slurry, and the interparticle forces caused by hydration were not considered here. According to the traditional colloid stability DLVO theory (Derjaguin, Landau, Verwey, and Overbeck’s theory), the micro-scale forces between particles in cement slurry mainly include van der Waals forces and electrostatic attraction. Among them, the van der Waals attraction between two particles with the same surface properties can be expressed by Equation (9) [34,35]:

(9) $$F_{vdw}≅\frac{-Aa^{*}}{12h^{2}}$$

where *A* is the non-retarded Hamaker constant, representing the distance between the particle and particle surface [36], and $a^{*}$ is the curvature radius of the particle. When the density of cement is 3.15 g/cm^3^, the Hamaker constant for cement is 1.6 × 10^−20^ J, and the curvature radius $a^{*}$ is in the order of 500 nm.

Additionally, the ions adsorbed on the particle surface can generate electrostatic forces [37], and the electrostatic repulsion between particles with the same surface potential can be expressed by Equation (10):

(10) $$F_{elect}≅-2\pi \varepsilon \varepsilon_{0}\Psi^{2}d\frac{Ke^{-Kh}}{1+e^{-Kh}}$$

where ε and $\varepsilon_{0}$ are the relative dielectric constant and vacuum dielectric constant, respectively; *d* is the particle radius; *Ψ* is the particle surface potential; K^−1^ is the Debye length. The relevant calculation parameters in the cement matrix are ε = 80 F·m^−1^, $\varepsilon_{0}$ = 8.85·10^−12^ F·m^−1^, *Ψ* = 5 mV, *d* = 5 μm, and *κ*^−1^ = 0.67 nm [35].

In the presence of polycarboxylate superplasticizers, due to their adsorption on the cement particle surface, a pronounced steric hindrance effect exists. The calculation formula for steric hindrance force is as follows:

(11) $$F_{ster}=\beta \left(\frac{5}{2^{1/3}}R_{AC}^{2/3}-\frac{1}{2}h^{-1/3}(3h+4(2^{2/3})R_{AC}({\frac{R_{AC}}{h})}^{2/3})\right)$$

where

(12) $$\beta =\left(\frac{2\pi k_{B}TR_{tip}}{\alpha }P^{-29/30}N^{-13/30}\right)$$

where $k_{B}$ is the Boltzmann constant, *k* = 1.380649 × 10^−23^ J/K, and $R_{tip}$ is the radius of the AFM cantilever tip, assumed to be semi-spherical. *N* is the number of monomers in each repeating unit of the comb-like polymer backbone, *P* is the number of monomers in each side chain, and the calculation formula for *α* is as follows:

(13) $$\alpha =\pi 2^{-3/10}a_{P}^{5/3}a_{N}{\left((1-2\chi )\frac{a_{P}}{a_{N}}\right)}^{2/15}$$

where $a_{P}$ is the size of the side chain unit, $a_{N}$ is the size of the main chain unit, χ is the Flory parameter, and $R_{AC}$ is the adsorption layer thickness of the comb-like polymer, with the following specific calculation formula:

(14) $$R_{AC}={\left(2\surd 2(1-2\chi )\frac{a_{P}}{a_{N}}\right)}^{1/5}a_{P}P^{7/10}N^{-1/10}$$

where the relevant calculation parameters for the steric hindrance force are $R_{tip}$ = 45 nm, $a_{N}$ = 0.25 nm, and $a_{P}$ = 0.36 nm. In 25 °C conditions, χ = 0.37, *P* = 23, and *N* = 3. It should be noted that the steric hindrance force expression given above refers to the interaction force between a sphere and a flat surface, while the steric hindrance force in the cement slurry involves the interaction between two spherical objects, and its curvature radius is half of the $R_{tip}$ value.

Under the influence of polycarboxylate superplasticizers, the forces between particles in cement slurry mainly consist of van der Waals forces, electrostatic attraction, and steric hindrance forces. After the addition of PAM to the cement slurry, due to the presence of its coagulation-forming action, it exhibits the “adsorption–lubrication–entanglement” action at different PAM concentrations. Among them, the adsorption bridging action of PAM can be represented by the so-called adsorption bridging force $F_{ads}$.

When the surface of cement particles is fully adsorbed, continuous addition of PAM will result in the presence of non-adsorbing polymer form. In this state, the conformational entropy loss cannot be compensated by adsorption energy; therefore, the polymer is excluded from the interface, resulting in the formation of a pure solvent region. When the distance between particles is smaller than the characteristic size of the polymer, the osmotic pressure difference between the polymer-depleted region and the bulk polymer solution leads to attractive interactions between particles, forming what is known as depletion attraction. This attractive energy *G_dep_* is numerically the energy required for two particles to separate from a distance of h to an infinite distance under osmotic pressure:

(15) $$G_{dep}=\Delta \Pi \Delta V$$

where ΔV is the additional volume that polymer molecules can occupy due to the overlap of the depletion regions of adjacent particles, and ΔΠ is the osmotic pressure difference between them. Considering a polymer solution as a dilute solution, the formula for the attractive energy in this case is as follows [23]:

(16) $$G_{dep}=-\frac{4\pi }{3}{\left(d+R_{g}\right)}^{3}(1-\frac{3r}{4\left(d+R_{g}\right)}+\frac{r^{3}}{16{\left(d+R_{g}\right)}^{3}})\rho kT$$

where *d* is the radius of cement particles; $R_{g}$ is the polymer’s radius of gyration; ρ is the number density of polymer molecules in the solution; *k* is the Boltzmann constant; *T* is temperature.

As the content of PAM continues to increase, when the concentration of PAM exceeds its characteristic concentration, the polymer exhibits significant entanglement. Using the average mesh size of this network flocculation structure as a parameter variable [23], the net attraction energy in the cement slurry can be calculated as shown in Equation (17):

(17) $$G_{net}≅-\frac{dkT}{\xi^{3}}{\left(\pi \xi -h\right)}^{2}$$

where *ξ* is the mesh size, calculated as follows:

(18) $$\xi ≅R_{g}{\left(\frac{\varphi }{\varphi^{*}}\right)}^{m}$$

For an ideal polymer solution, *m* = −3/4, where φ is the volume fraction of the polymer in the bulk solution, and $\varphi^{*}$ is the critical overlap volume fraction of polymer coils in the solution. For a given polymer, the relevant length is proportional to *c*^−3/4^ [23].

#### 2.3.2. Evolution Trend of Microscopic Cohesive Forces

In the comparative solution of this experiment (PAM = 0.0%), the forces between pure solution particles are the sum of van der Waals forces, electrostatic attraction, and steric hindrance:

(19) $${F=F}_{vdw}+F_{elect}+F_{ster}$$

Due to the significant presence of polycarboxylate water-reducing agents in the cement slurry, the steric hindrance force is much larger compared to the other two forces. According to reference [32], with a water-to-cement ratio of 0.5 and a specific surface area of 367.9 m^2^/kg for cement particles, the interparticle spacing h is approximately 2.5 μm. Substituting into Equation (19), the magnitude of the internal forces within the cement slurry when PAM is at 0.0% is in the order of 5.0 × 10^−7^ N.

After adding PAM to the cement slurry, due to the presence of adsorption bridging forces, the adsorption interactions between particles first balance the repulsive effects caused by steric hindrance, and then, the forces between particles start to be dominated by adsorption. Although the exact magnitude of the adsorption force is difficult to calculate, it is clear that PAM primarily adsorbs on the surface of cement particles through hydrogen bonding and complexation, bridging multiple cement particles together. These various adsorption interactions are mutually cross-coupled, resulting in the increment of the adsorption bridging force being greater than the single power of the actual adsorption amount of the particle, i.e.,$F_{bri}\propto {c_{sd-P}}^{>1}$. Through total organic carbon experiments, it was found that the PAM content is related to its adsorption in the power of 6.6. Therefore, the relationship curve between the adsorption bridging force and polymer content is as follows:

(20) $$F_{ads}\propto {c_{sd-P}}^{>1}\propto c^{>0.30}$$

As the cement particle surfaces gradually become saturated with adsorption, the depletion attractive force gradually becomes the main form of interaction, as described by Equation (16). In an ideal dilute solution, when $h\ll 2R_{g}$, the polymer’s number density is proportional to the ratio of polymer concentration to molecular mass ($\rho \propto \frac{c}{M}$), while *R_g_* is proportional to the 3/5 power of the polymer’s molecular mass ($R_{g}\propto M^{3/5}$), and thus, the relationship between depletion attraction and polymer concentration is as follows:

(21) $$F_{dep}\propto \rho R_{g}\propto \frac{c}{M}M^{3/5}\propto cM^{-2/5}$$

Considering the fixed molecular mass of the polyacrylamide used in this study, the depletion attraction force is then directly proportional to the concentration of PAM.

When the PAM content exceeds the critical concentration, that is, when the PAM net-pulp is in a semi-diluted solution state, considering *h* ≪ *πξ*, as discussed earlier with $\xi \propto c^{-3/4}$, the relationship between the mesh attraction force and polymer concentration becomes the following:

(22) $$F_{net}\propto \frac{1}{\xi }\propto c^{3/4}$$

**Figure 15:**
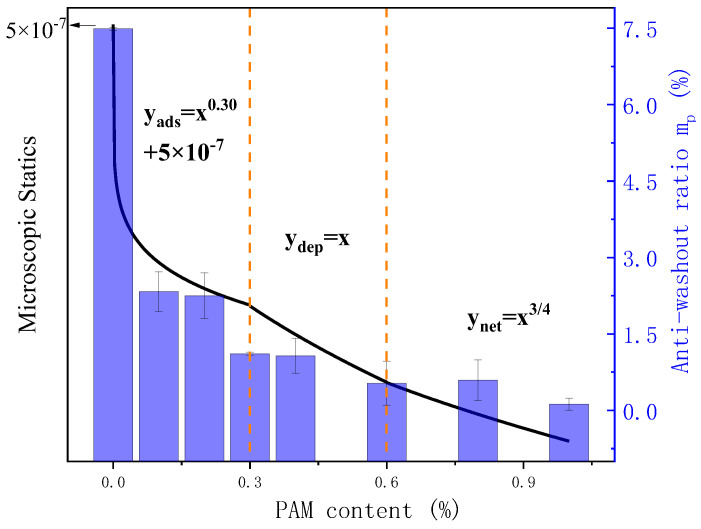
Total microscopic statice and anti-washout ratio of PAM paste.

Based on the above analysis, the predicted curves of the microscopic interactions between suspended particles under different forms of PAM action, along with the measured mass loss rate characterizing the net slurry anti-dispersion performance, are illustrated in Figure 15. The results demonstrate a high consistency between the growth trend of the microscopic interactions between cement particles in the PAM net pulp, calculated from micro-rheological parameters and the flocculation mechanism model, and the actual macroscopic mass loss situation. In other words, the interactions between cement particles within the cement slurry can directly characterize the underwater anti-dispersion performance. As the cohesive attraction between particles increases, their ability to withstand the impact of external water flow becomes stronger, leading to improved resistance against erosion and dispersion.

When the PAM content is below 0.3%, the primary force between particles is the adsorption force. The long-chain structures quickly adhere to the charged cement particle surfaces, resulting in an increase in the adsorption force within the suspension system. The adsorption amount approximately follows a power function of 0.30 with respect to the polymer increment, leading to an enhanced macroscopic anti-dispersion performance. As the saturation value of particle free surfaces is reached, the depletion attraction force induced by osmotic pressure difference gradually becomes dominant. The numerical value of depletion attraction is linearly positively correlated with the polymer increment, which continuously improves the anti-dispersion performance of the suspension. When the PAM content exceeds the critical concentration, the depletion attraction force transitions to a steric hindrance force, which is proportional to the power of 3/4 of the PAM content. At this stage, the suspension’s anti-dispersion performance has already reached a higher level, and the magnitude of improvement in anti-dispersion performance diminishes.

When the PAM content is more than 0.3%, the dominant effect is the adhesion force between particles. The long-chain structures quickly adhere to the charged cement particle surface, with the adsorption amount approximately proportional to the 0.30 power of the polymer increment. This leads to a rapid increase in the adhesion force between particles in the suspension system, resulting in improved macroscopic anti-washout resistance. However, at this stage, the saturation adsorption point has not been reached. There are still some free particles in the suspension, which results in poor dispersion resistance of the slurry. As the free surface of the particles reaches saturation, the dominant effect gradually shifts to the depletion attraction. This attraction force is linearly positively correlated with the polymer increment. Under this condition, the dispersion resistance of the slurry gradually increases, and the corresponding fluidity also slightly improves due to the lubricating effect of the polymer. Until the PAM content reaches the critical concentration, the dispersion resistance slurry achieves its optimal performance. Further increasing the PAM content can enhance its dispersion resistance, but it significantly reduces the flowability of the slurry due to the entanglement effect, resulting in poorer overall workability of the slurry.

## 3. Conclusions

Based on the experimental results, the main conclusions of this study can be summarized as follows:

1. As PAM content increases, the zeta potential of the OPC slurry firstly rises (from −4.21 mV to −9.94 mV), then falls (to −8.06 mV), and finally rises again (to −11.57 mV). Different PAM concentrations exhibit varying effects in the solution;
2. Fourier transform infrared spectroscopy confirmed interactions between metal cations and COO- groups as well as hydrogen bonding between -NH_2_ groups and water. These interactions drive the adsorption of PAM’s long chains onto cement particles;
3. Total organic carbon tests showed that PAM adsorption on cement particles peaks at around 0.3%, reaching saturation. The critical concentration indicates that the polymer winding application point occurs at 0.67% PAM content;
4. With increasing PAM concentration, the polymer long chains follow an “adsorption–lubrication–entanglement” model, which explains changes in rheological parameters. This finding also applies to CSA cement solutions;
5. Microscopic static analysis revealed steric hindrance, depletion attraction, and net forces in the PAM–slurry system, demonstrating that the polymer’s gelling force effectively indicates its resistance to water erosion.

## 4. Materials and Methods

### 4.1. Materials and Protocol

This study employed ordinary Portland cement (OPC) and sulfoaluminate cement (CSA) as the cementitious materials, in accordance with the standards of General-Purpose Portland Cement [36] and Sulfoaluminate Cement [37]. The chemical composition of the cement was determined using the Panalytical Axios Fast X-ray fluorescence spectrometer (PANalytical B.V., Eindhoven, the Netherlands), and the results are presented in Table 1. The primary chemical components include CaO, SiO_2_, Al_2_O_3_, Fe_2_O_3_, and SO_3_ as well as minor amounts of MgO, K_2_O, and Na_2_O. Specifically, ordinary Portland cement has a CaO content of 66.65%, while calcium sulfoaluminate cement exhibits an Al_2_O_3_ content of 28.17% and SO_3_ content of 12.01%.

**Figure 16:**
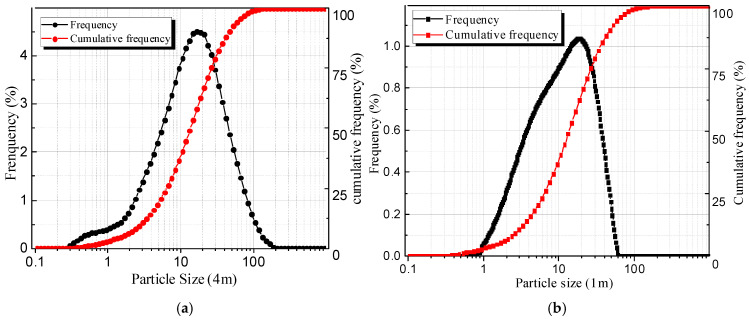
Particle size distribution of OPC and CSA cement: (**a**) OPC; (**b**) CSA.

The particle size distribution of cement particles was analyzed using the dry powder multi-point sampling method with the Bettersize 2000 laser particle size analyzer (Bettersize Instruments, Dandong, Liaoning, China), as illustrated in Figure 16 In the case of OPC cement, the particle size frequency distribution exhibited D10, D50, and D90 values of 2.987 μm, 14.50 μm, and 49.18 μm, respectively, yielding a specific surface area of 367.9 m^2^/kg. Similarly, the particle size frequency distribution for CSA cement indicated D10, D50, and D90 values of 2.75 μm, 11.10 μm, and 31.60 μm, respectively, together with a specific surface area of 923.5 m^2^/kg.

**Figure 17:**
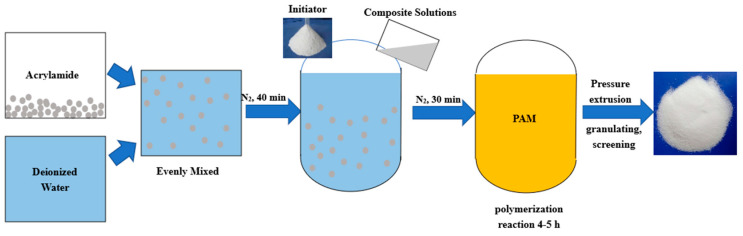
Process diagram of polyacrylamide gel.

For the anti-washout cement paste, a non-ionic polyacrylamide polymer (Shengyu Co., Ltd., Nanjing, Jiangsu, China) with a molecular weight of 10 million Dalton and PCA^®^-Type I (SBT, Nanjing, Jiangsu, China), which is known for its effectiveness as a polycarboxylic acid water reducer, were incorporated additives in our research. The specific aggregation process of PAM gel is shown in Figure 17 and is as follows: (1). Mix acrylamide (AM) and deionized water in a ratio of 1:5 according to the formula, and transfer to the dissolution tank. Stir until the raw materials are thoroughly and uniformly mixed. (2). After cooling the dissolution tank, pump the mixture into the reaction kettle for the polymerization reaction. (3). After purging with nitrogen for 40 min, add the initiator, azobisisobutyronitrile (AIBN), along with other composite solutions prepared with chain transfer agents, complexing agents, reducing agents, a first-stage oxidant, a second-stage oxidant, and auxiliary reducing agents. (4). After continuously introducing nitrogen for 30 min, stop and let the polymerization reaction proceed for 4–5 h to obtain the polymer colloid. (5). Open the compressed air inlet valve and use compressed air at 0.2 to 0.3 MPa to push out the material, completing the discharge in about 30 min. (6). Finally, proceed with cutting, granulating, drying, crushing, and screening to obtain non-ionic polyacrylamide.

To maintain the desired consistency, a water/cement ratio of 0.5 was employed. The addition of the polycarboxylic acid superplasticizer, following the guidelines outlined in the Quality Standard and Test Method of concrete water-reducing agent [38], was set at 2.0% of the cement mass [9]. To study the impact of the polymer’s action mechanism, different concentrations of PAM, ranging from 0.0% to 1.2%, were incorporated, as per the previous study [38].

### 4.2. Testing Methods

#### 4.2.1. Zeta Potential Testing

**Figure 18:**
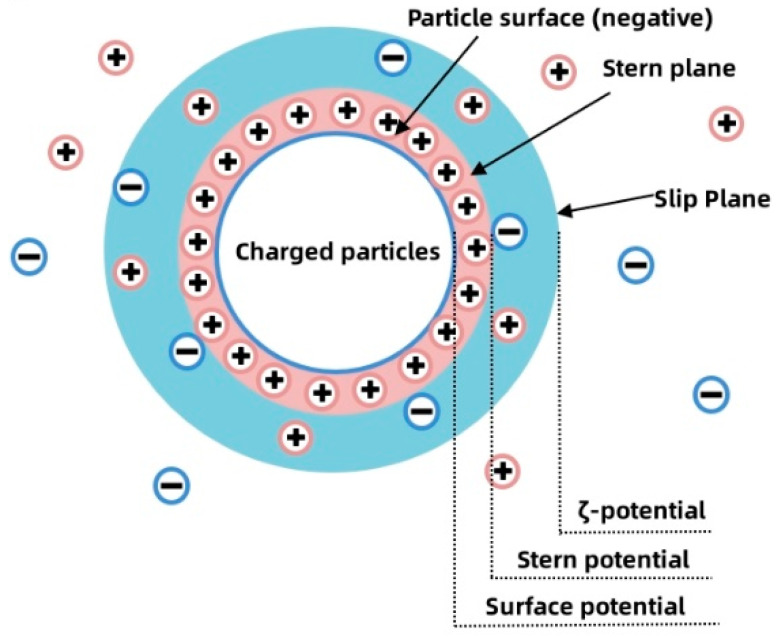
Double Electron Layer Theory for cationic particle.

The concept of cationic particle zeta potential is showcased in Figure 18. Electrophoretic light scattering combines laser light scattering with microelectrophoresis [39]. When a laser beam passes through the electrophoresis cell, the Brownian motion of cement particles in the cell causes shifts in laser frequency and phase. These shifts are directly proportional to the velocity of charged particles and can be used to determine particle electrophoretic velocity and subsequently calculate the zeta potential value. Conforming to the ISO-13099-2 standard [40], electrophoretic light scattering was employed for zeta potential testing of PAM slurry. Since the previous research has established that 0.6% PAM is its optimal dosage [9,21], PAM content gradients of 0.0%, 0.3%, 0.6%, and 0.9% by cement mass were tested for their effects on the change in slurry potential. A well-mixed PAM slurry was placed in the Malvern Zetasizer Nano ZS 90 analyzer (Malvern Panalytical, Shanghai, China) and stirred at 350 r/min for 2 min before measurement, and each composition was subjected to three repeated tests to obtain accurate data.

#### 4.2.2. Fourier Transform Infrared Spectroscopy

To eliminate the early hydration effects of cement and preserve the initial chemical bonding state of the material, freshly mixed PAM slurry was placed in a 10.0 mL test tube and rapidly frozen using liquid nitrogen. The material was then subjected to freeze drying for a minimum of 24 h using the BK-FD10S vacuum freeze dryer (GaoXin Biological Sensor Research Institute Co., Ltd., Jinan, China). Bromide potassium (KBr) powder was mixed with the dried slurry sample in a 100:1 ratio to complete the experimental preparation stage. For further analysis, Fourier transform infrared spectroscopy (FTIR) was carried out using the Boen 29865 spectrometer (Feierbon Precision Instruments Co., Ltd., Shanghai, China). The spectroscopy range was set from 4000 cm^−1^ to 100.0 cm^−1^, with a resolution of 4.0 cm^−1^. Each FTIR test consisted of a minimum of 16 scans, and the test was performed once. To ensure accurate and reliable data, three repeated FTIR tests were conducted for each given ratio of non-dispersed underwater slurry to determine the optimal spectrum.

#### 4.2.3. Total Organic Carbon Test

The total organic carbon (TOC) test was conducted by acidifying the sample to measure its total carbon and inorganic carbon content and then calculating the organic carbon content based on the difference between the two values [1]. Specifically, 200.0 g of cement, 100.0 g of water, 2.0 g of polycarboxylate superplasticizer, and the corresponding concentration of polymer solution were thoroughly mixed and stirred at high speed. For varying ratios of PAM slurry, the mixture was centrifuged using the Riebeck M1324R centrifuge (Ruiwode Life Technology Co., Ltd., Shenzhen, China) at a speed of 5000 r/min for 5 min, and the upper clear liquid (approximately 9.0 g) was collected. Diluted HCl (1 mol/L) solution was added to eliminate the presence of inorganic carbon substances [14]. After that, the TOC content of the sample solution was determined using the XPERT-TOC/TNb total organic carbon analyzer (Trace Elemental Instruments, BC Delft, The Netherlands). The relative adsorption rate of the PAM gel can be calculated using the following formula:

(23) $$R_{a}=\frac{{TOC}_{P}+{TOC}_{C}-{TOC}_{P+C}}{{TOC}_{P}+{TOC}_{C}}$$

where $R_{\;a}$ represents the relative adsorption rate of the PAM gel at the corresponding PAM dosage; ${TOC}_{P}$, ${TOC}_{C},\;\mathrm{and}\;{TOC}_{P+C}$ are the total organic carbon contents of the PAM pure solution, the reference slurry, and the slurry with the corresponding PAM content, respectively.

## Figures and Tables

**Table 1:** Chemical composition of OPC and CSA cement (unit: %).

Compositions	CaO	SiO_2_	Al_2_O_3_	Fe_2_O_3_	SO_3_	MgO	K_2_O	Na_2_O	Others
OPC	66.65	17.88	6.35	3.72	2.66	0.61	0.85	0.12	1.16
CSA	46.73	8.14	28.17	2.48	12.01	0.81	0.23	0.000	1.43

Note: The loss on ignition for OPC and CSA cement is 1.35% and 1.73%, respectively.

## Data Availability

The raw data supporting the conclusions of this article will be made available by the authors on request.
